# 1819. The Impact of Vascularized Lymph Node Transplant in Reducing the Rate of Cellulitis in Patients with Breast Cancer–Related Lymphedema

**DOI:** 10.1093/ofid/ofad500.1648

**Published:** 2023-11-27

**Authors:** Alejandro Perez, Mark Schaverien, Monica George-Palop, Carlos Barcenas, George M Viola

**Affiliations:** The University of Texas at MD Anderson Cancer Center, Houston, Texas; The University of Texas at MD Anderson Cancer Center, Houston, Texas; The University of Texas at MD Anderson Cancer Center, Houston, Texas; The University of Texas at MD Anderson Cancer Center, Houston, Texas; The University of Texas MD Anderson Cancer Center, Houston, Texas

## Abstract

**Background:**

Lymphedema following breast cancer surgery is a chronic and disabling complication that may lead to recurrent cellulitis. Innovative advances in treatment of breast cancer–related lymphedema have evolved beyond conservative management to include vascularized lymph node transplant (VLNT). Herein, we analyzed the impact of VLNT in reducing the rate of upper extremity cellulitis in breast cancer survivors.

**Methods:**

The charts of all patients who had breast cancer and mastectomy, whose course was complicated by upper extremity lymphedema, and who ultimately underwent VLNT at our comprehensive cancer center from 2017 to 2021 were reviewed. Patients who had at least one episode of cellulitis within the year prior to VLNT and were followed for 24 months were included. Thereafter, we reviewed patients’ demographics, cancer management, breast reconstructive procedures, VLNT surgeries, and infectious complications.

**Results:**

We included 66 patients in our study. Their median age was 57 years (IQR, 23-76 years) (Table 1). The majority were White (88%), with a mean (± SD) body mass index of 29.4 ± 6.7 kg/m^2^. Most patients had invasive ductal carcinoma (82%) and received chemotherapy (94%), including taxane-based regimens (85%), which increases the risk for lymphedema. Also, most patients received radiation therapy (86%), including to the axillary nodal basin (92%). Furthermore, most underwent unilateral mastectomy (74%) with axillary lymph node dissection (95%) followed by autologous or implant-based reconstruction (both 42%) (Table 2). VLNT was performed at a median of 92 months (IQR, 32-156 months) after mastectomy and were mainly harvested from the inguinal and gastroepiploic regions (both 32%). After VLNT, 58 (88%) patients remained infection-free throughout the 24-month follow-up period. The only factor associated with recurrent cellulitis was the need for a second lymph node transfer or lymphovenous bypass surgery, likely owing to a significant lack of lymphedema reduction (p=0.006).

**Figure:**
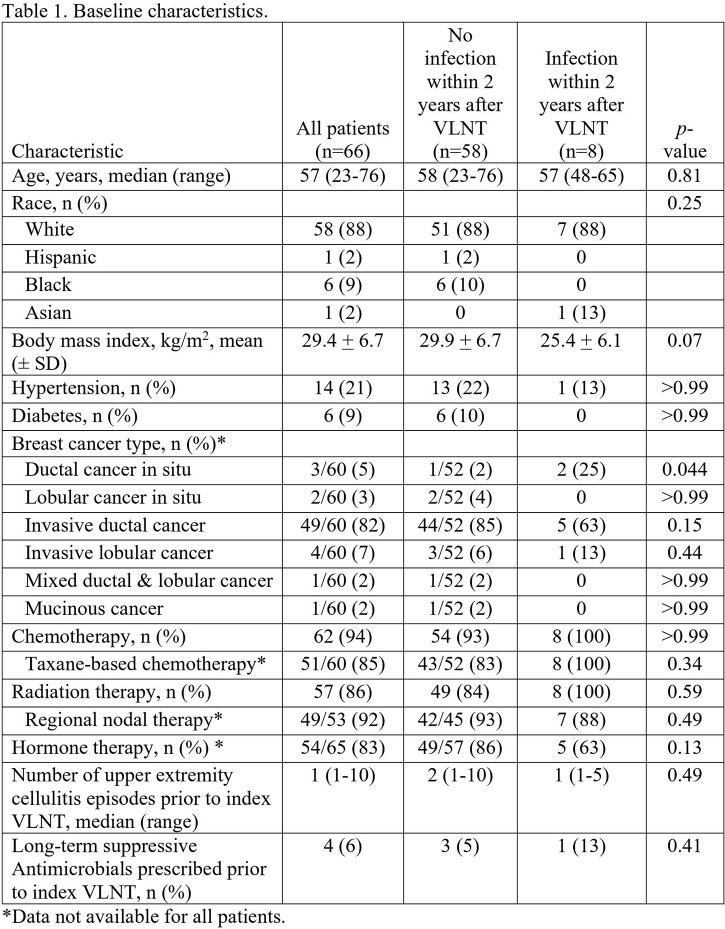

**Figure:**
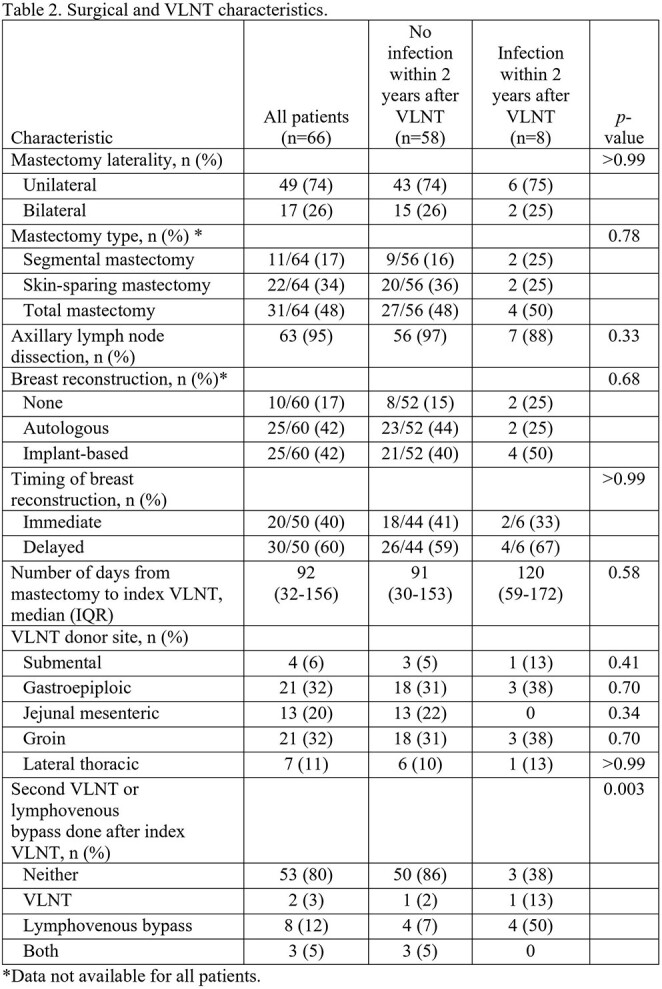

**Conclusion:**

The novel approach of VLNT in the management of breast cancer–related lymphedema is associated with significantly decreased recurrent cellulitis rates and should be considered as part of the infectious diseases treatment armamentarium.

**Disclosures:**

**All Authors**: No reported disclosures

